# A Unique Automation Platform for Measuring Low Level Radioactivity in Metabolite Identification Studies

**DOI:** 10.1371/journal.pone.0039070

**Published:** 2012-06-18

**Authors:** Joel Krauser, Markus Walles, Thierry Wolf, Daniel Graf, Piet Swart

**Affiliations:** 1 DMPK/Biotransformation Laboratories, Novartis Pharma AG, Basel, Switzerland; 2 DMPK/Isotope Laboratories, Novartis Pharma AG, Basel, Switzerland; Genentech, United States of America

## Abstract

Generation and interpretation of biotransformation data on drugs, i.e. identification of physiologically relevant metabolites, defining metabolic pathways and elucidation of metabolite structures, have become increasingly important to the drug development process. Profiling using ^14^C or ^3^H radiolabel is defined as the chromatographic separation and quantification of drug-related material in a given biological sample derived from an *in vitro*, preclinical *in vivo* or clinical study. Metabolite profiling is a very time intensive activity, particularly for preclinical *in vivo* or clinical studies which have defined limitations on radiation burden and exposure levels. A clear gap exists for certain studies which do not require specialized high volume automation technologies, yet these studies would still clearly benefit from automation. Use of radiolabeled compounds in preclinical and clinical ADME studies, specifically for metabolite profiling and identification are a very good example. The current lack of automation for measuring low level radioactivity in metabolite profiling requires substantial capacity, personal attention and resources from laboratory scientists. To help address these challenges and improve efficiency, we have innovated, developed and implemented a novel and flexible automation platform that integrates a robotic plate handling platform, HPLC or UPLC system, mass spectrometer and an automated fraction collector.

## Introduction

Generation and interpretation of biotransformation data on drugs, i.e. identification of physiologically relevant metabolites, defining metabolic pathways and elucidation of metabolite structures, have become increasingly important to the drug development process as well as more visible to health authorities (HA). Since the introduction of the FDA metabolite in safety testing “MIST” guidance and the International Conference on Harmonization (ICH) metabolite guidance M3, early information on metabolites structures and their systemic exposure is of high relevance [1], [2].

Profiling using ^14^C or ^3^H radiolabel is defined as the chromatographic separation and quantification of drug-related material in a given biological sample derived from an *in vitro*, preclinical *in vivo*, or clinical study. Initial metabolite profiling data from biotransformation studies are often generated in a high throughput manner during the discovery phase of pharmaceutical development. However, this data has limitations as it is semi-quantitative in nature and is based mostly on mass spectrometric response factors [3], [4]. Metabolite profiling data generated during early and late stage development phase requires accurate methods of quantification of metabolites in circulation and excreta. The profiled chromatographic peaks are also characterized structurally using analytical techniques such as mass spectrometry or in some cases even NMR. Thus, linking the structural data with the profiled data, a metabolism pathway can be elucidated on a quantitative level.

Radiolabeled (^14^C or ^3^H-labeled) compounds can be used for accurate quantification, and offer advantages that eliminate the need for sample calibration curves as well as in certain cases providing a signature isotope distribution pattern (^14^C) that aids in the MS identification of metabolites. Chromatographically separated metabolites which contain the radiolabel derived from the parent compound are quantitated through radioactive decay (β-emission). Radioactive decay can either be counted (detected) using on-line scintillation radioflow detectors (RFD) or off-line microplate scintillation counters (MSC), which count fractionated samples using well plate format, e.g. Topcount [5]–[7].

For preclinical *in vivo* or human ADME, international guidance set clear limits on radiation burden and exposure levels [8]. In the cases where low levels of radioactivity must be administered, the on-line radio detection method often does not provide sufficient detection sensitivity and quantification (lower limit of quantification (LLOQ): 100–500 dpm or 0.8–4 pmol) [9]. Thus, off-line counting is necessary due to the requirements of a much lower (LLOQ: 2–5 dpm or 16–40 fmol), which is achieved by a much higher signal to noise ratio generated from longer data acquisition times than compared to on-line counting [9]. In context of a collected chromatographic peak over separate wells, off-line detection would be in the range 10–20 fold more sensitive than on-line detection.

Metabolite profiling by conventional HPLC often requires long chromatographic run times ranging between 90 to 120 min per sample injection. High-performance liquid chromatography (HPLC) coupled to RFDs is an established and robust method to separate and detect radiolabeled drug metabolites. Recent chromatography developments that favor smaller particle chromatography (<2 μm) has led to increased use of ultra-performance liquid chromatography (UPLC).

**Figure 1:**
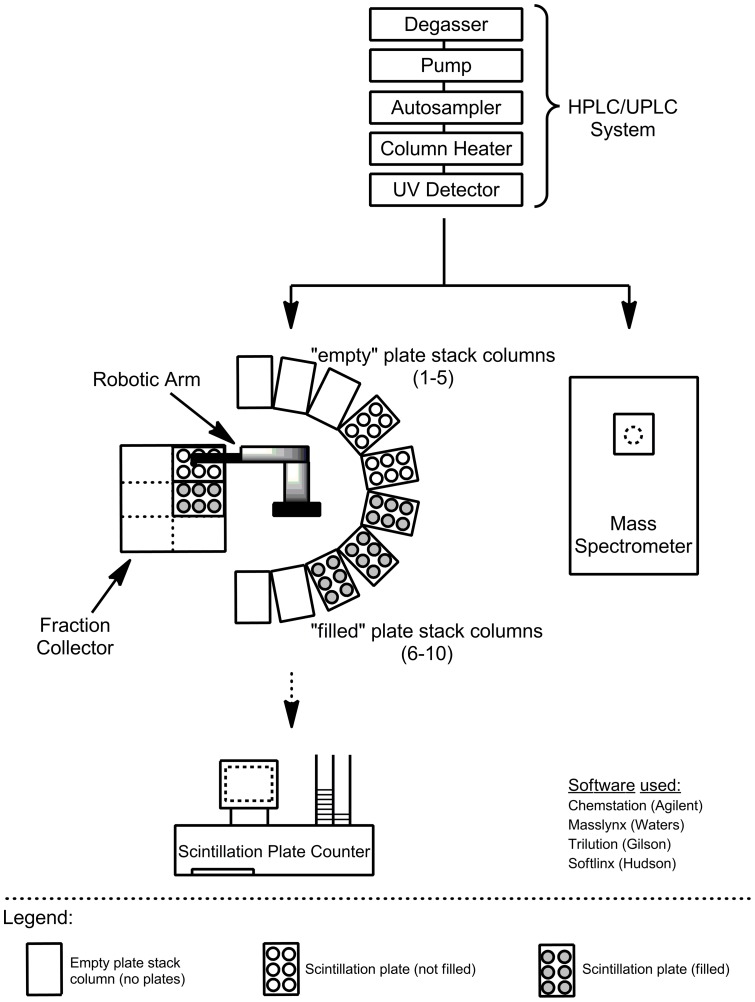
Schematic of the interfaced instruments: HPLC (Agilent)/UPLC (Waters), robotic plate handler (PlateCrane) robot, fraction collector (Gilson) and mass spectrometer (Waters).

UPLC coupled with high resolution and fast scanning mass spectrometers has been demonstrated to be a robust, efficient for drug metabolite separation and identification [6], [7]. However, UPLC coupled with RFDs has significant limitations for metabolite quantification, which arises from resolution and sensitivity loss due to the eluant-scintillant mixing processes occurring in the flow cell [5].

**Figure 2:**
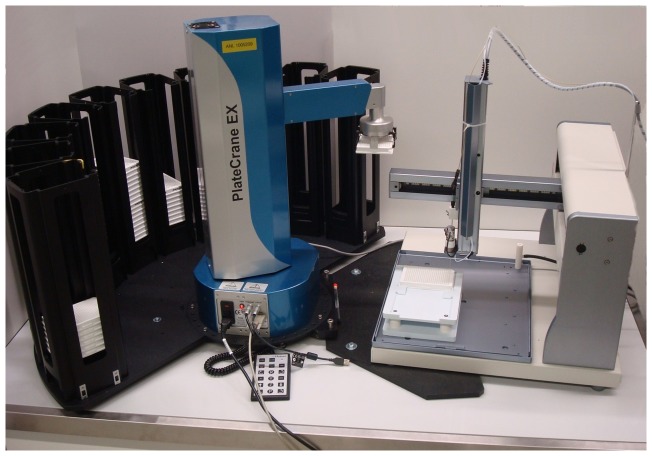
The robotic plate handler coupled to fraction collector.

**Figure 3:**
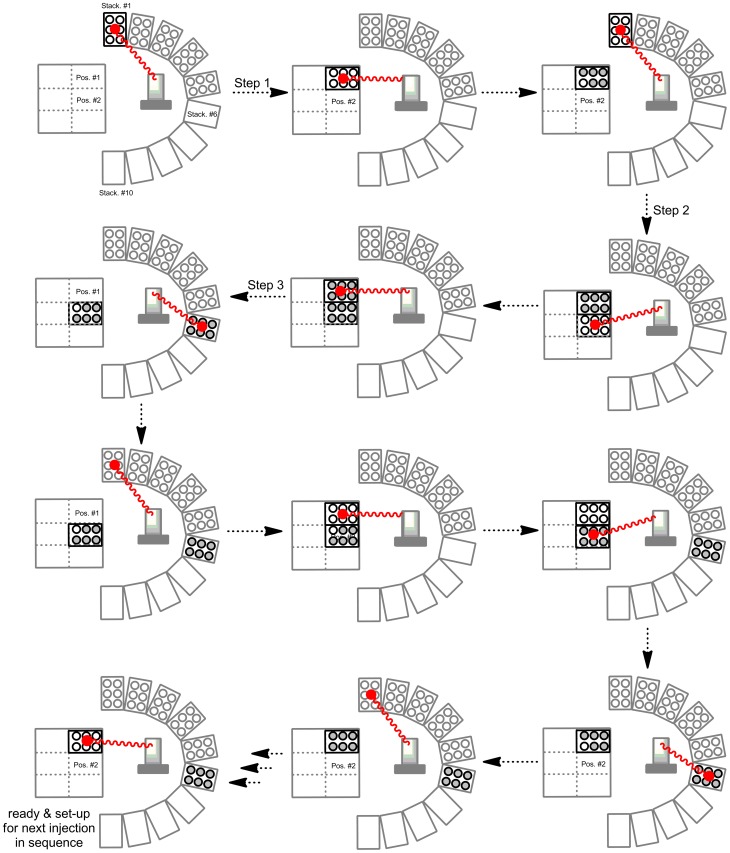
Plate movement sequence by robotic plate handler for a single chromatographic run.

**Figure 4:**
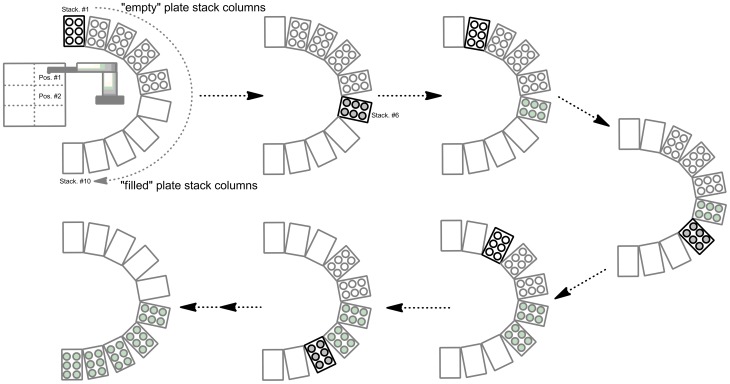
Plate stacking sequence by robotic plate handler for a programmed sequence of chromatographic runs.

**Figure 5:**
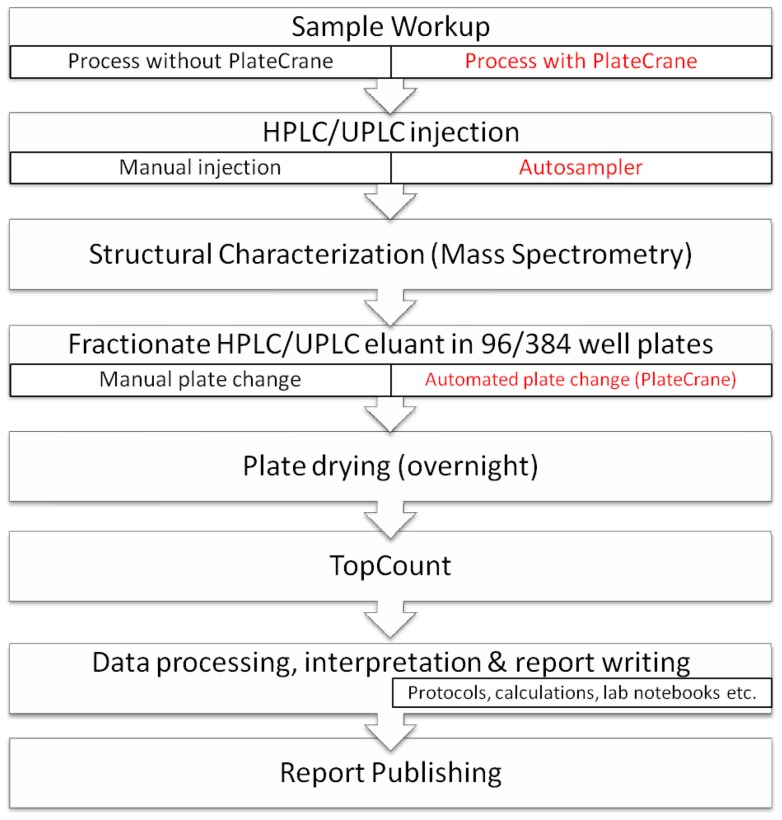
Sequential workflow for sample processing and analysis, from initiation to completion.

**Table 1:** Calculated time for current and streamlined processes.

	Without PlateCrane		With PlateCrane	
Step in process	hours	WD	hours	WD
Sample preparation	8	2	8	2
Chromatographic run time	8	4	24	1 ^a)^
Plate drying	8	2 ^b)^	24	1 ^c)^
TopCount measurements	24	4.5 ^d)^	24	4.5 ^d)^
**Total time**		**12.5**		**8.5**

* Assumption: 10 samples, 8 collected plates per sample and a chromatographic run time of 90 minutes.

hours – # work hours per day; WD – # work days required for each step.

**Figure 6:**
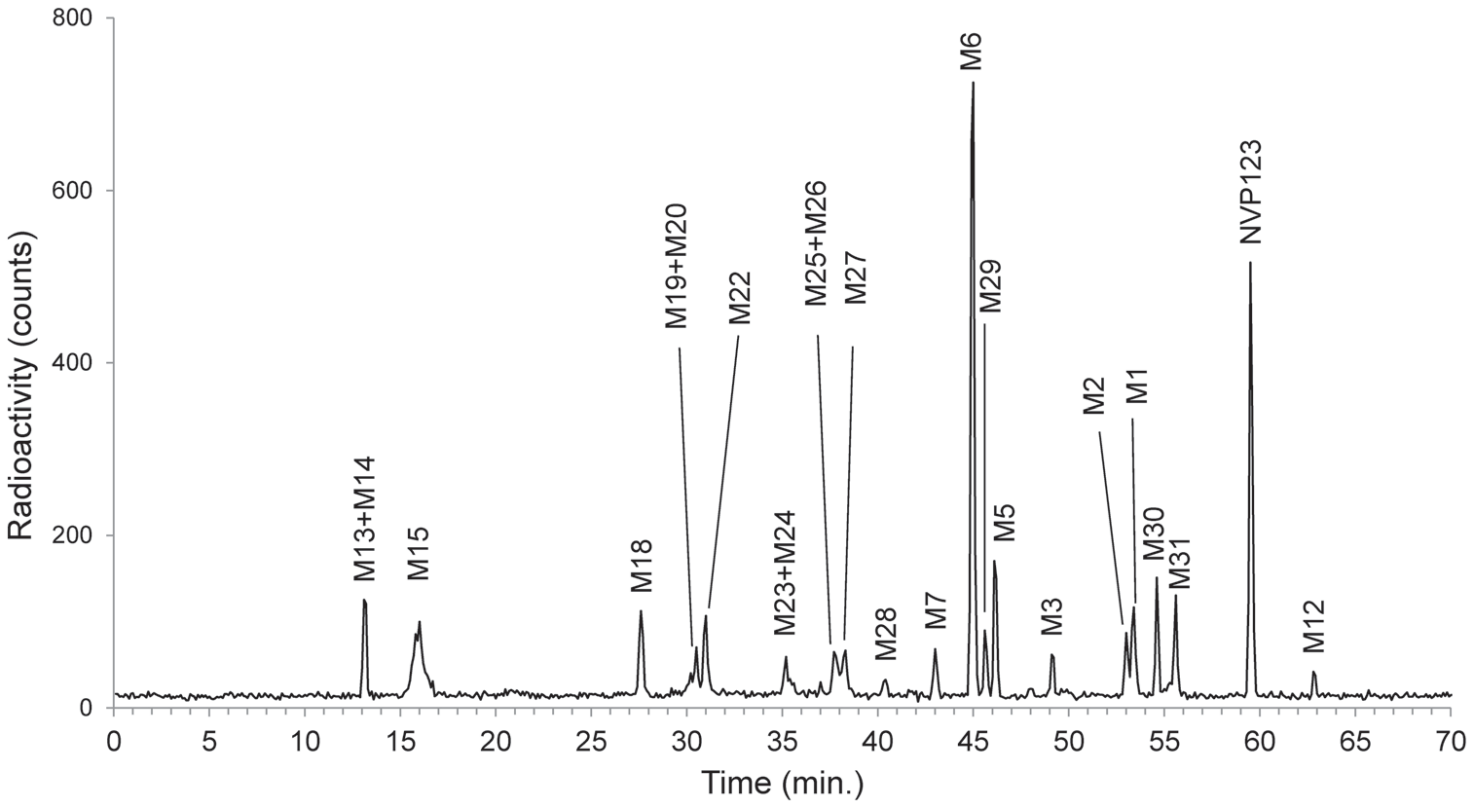
Reconstructed HPLC radiochromatogram of [^14^C]-NVP123 and its metabolites from human plasma. Note, “M#” (e.g. M13) denotes a designated metabolite number, which is not assigned based on order of chromatographic elution.

**Figure 7:**
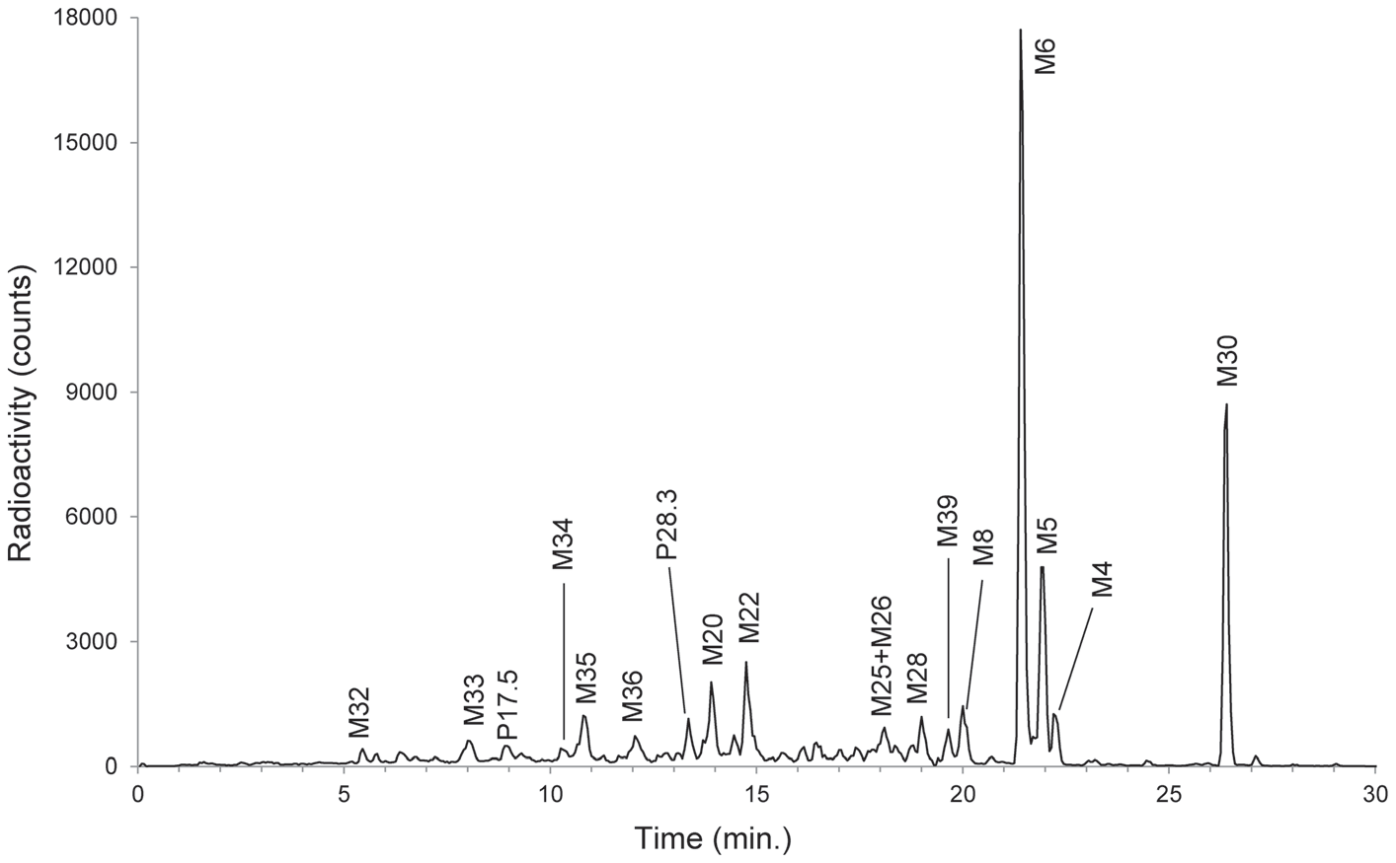
Reconstructed UPLC radiochromatogram of [^14^C]-NVP123 and its metabolites from dog urine. Note, “M#” (e.g. M13) denotes a designated metabolite number, which is not assigned based on order of chromatographic elution.

**Figure 8:**
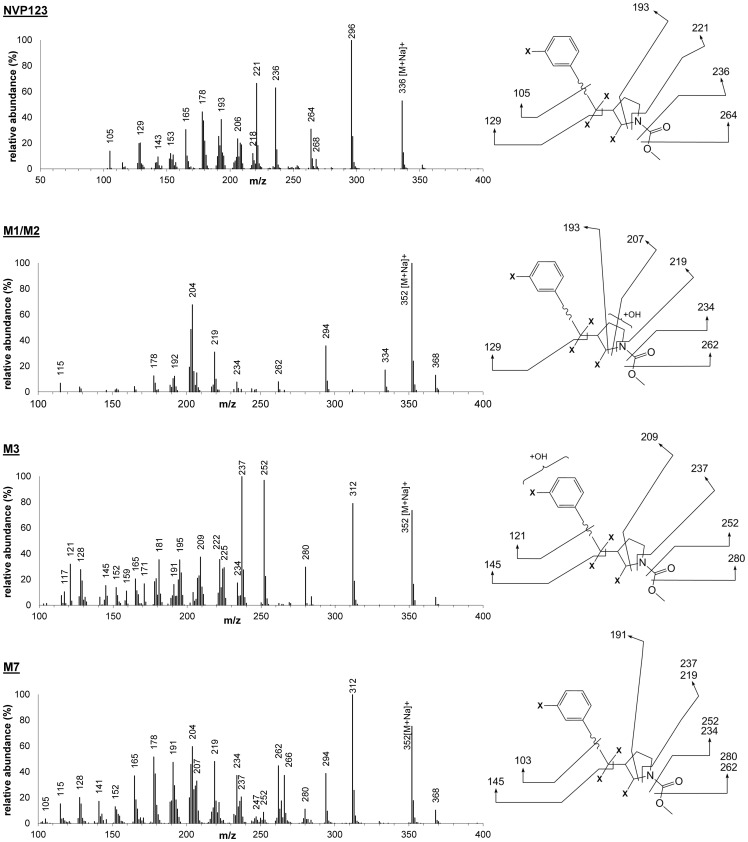
Representative mass spectrometric structure elucidation of [^14^C]-NVP123 and its resulting metabolites M1/M2, M3 and M7. (**x** in structure denotes truncation).

**Figure 9:**
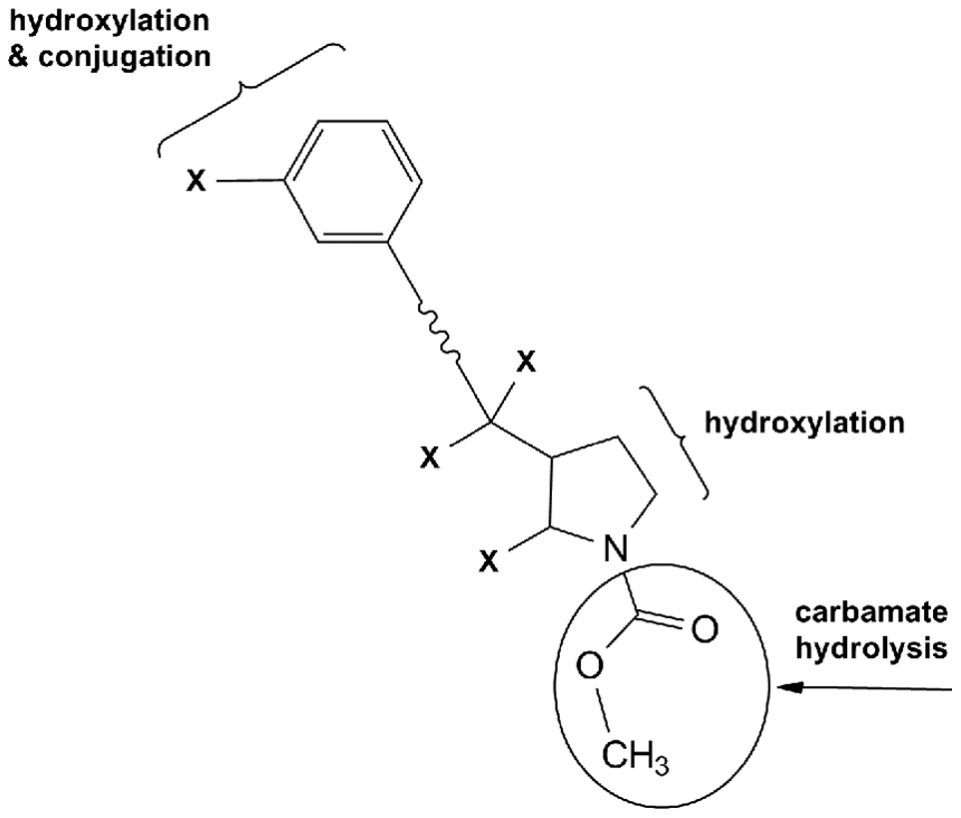
Overview of representative biotransformation reactions for [^14^C]-NVP123. (**x** in structure denotes truncation).

UPLC coupled with MSC, e.g. TopCount, has been successfully applied to both identification and quantification of metabolite while preserving chromatographic resolution [5], [10]. This combination offers advantages of higher sensitivity and resolution compared to the traditional on-line approach, i.e. HPLC with RFDs. Specifically, the narrow peak widths and longer counting times allow accurate profiling and measurement of samples containing low concentrations of drug-related material. Unlike RFDs, MSC systems are not limited by residency times and allow longer counting times. Representative counting times for MSC are 2–5 min per well compared to RFD cell counting times of 7–10 s. For MSC, there is a trade-off for resolution versus sensitivity, i.e. increasing the number of fractionated wells will increase resolution, but will in turn result in lower sensitivity. MSC is a robust means of reliable signal detection for high efficiency UPLC separations.

Some limitations exist for the HPLC/UPLC coupled to a MSC. One limitation is the inherent delay between sample injection and data viewing and processing. Another limitation is the longer counting durations required for sample measurements, which can serve either as an advantage or a disadvantage. To further reduce analysis times, microplate imagers (Viewlux) have been shown to reduce data acquisition times for 384 well plate counting in comparison to TopCount [11]. Lastly, manual plate handling and changing prior to scintillation counting still remains a very time intensive step even though chromatographic run times may be significantly reduced using UPLC with off-line radioactivity microplate imagers. Assuming HPLC run times of 90 minutes, only 2–3 samples could be processed per day as the collected fraction plates must be constantly changed manually.

To address the manual plate handling limitation and automate the process to increase throughput, we developed and implemented a novel automation technology by linking two separate technologies designed for two different applications. Specifically, a robotic plate handling platform was linked to a fraction collector, a HPLC or UPLC with UV detection, and a mass spectrometer.

## Materials and Methods

### Materials

Solvent and chemical materials used for this project were Uvasol™ spectrometry grade acetonitrile (Merck, Darmstadt, Germany), LC-MS grade water (Fisher Scientific, Leicestershire, UK), formic acid puriss p.a.; ∼98% (T) (Fluka Analytical, Germany) and ammonium formate Ultra ≥99.0% (NT) (calc. based on dry substance) (Fluka Analytical, Germany). The scintillation material used for this project was LumaPlates yttrium silicate scintillation-coated 96 well plates (Packard BioScience, Groningen, Netherlands).

Biological materials used for this project were human plasma and dog urine. Human plasma samples were taken from healthy volunteers after administration of a Novartis development compound (NVP123) which was in accordance to study protocol. The clinical part of the study was performed in accordance with Good Clinical Practice guidelines and the Declaration of Helsinki (1964 and subsequent revisions). All subjects gave written informed consent before entering the study. The protocol and the radiosafety assessment were approved by the local Basel, Switzerland ethics committee, and the Swiss federal Office of Public Health (Radioprotection Division in Bern, Switzerland), respectively. With Owner's and Study Director's consent, dog urine samples were taken in accordance with international guidelines and Swiss law for animal welfare. Specifically, all experiments were carried out in accordance with authorization guidelines of the Swiss Federal and Cantonal veterinary offices for care and use of laboratory animals. The animal experimental authorization number is “17 (17-Nov-2008) Kantonales Veterinäramt Basel”.

### Sample Preparation

The absorption, distribution, metabolism and excretion of [^14^C]-NVP123 were investigated in four healthy male volunteers after a single oral dose of 200 mg with a radioactive dose of 1.61 MBq. The whole body radiation dose estimation was less than 1.0 mSv.

Metabolite profiling was determined in plasma, urine and feces matrices. For plasma, individual samples of subjects taken at the same time post-dose were analyzed to establish the pharmacokinetics. For feces and urine, each subject was pooled across the collection period of 0–96 hours. Sample workup and processing in each matrix, e.g. extraction, drying and reconstitution, are described in the following paragraphs. Note, the dog urine sample was collected, processed and analyzed in a similar manner as described for the human urine below. In total, 300 [^14^C]-NVP123 samples (preclinical and clinical) were collected and analyzed using this automation platform.

Plasma aliquots (4 mL) were weighed in 50 mL BD FalconTM tubes and extracted with 30 mL of acetonitrile (UvasolTM Merck, Darmstadt, Germany). Samples were stirred, chilled for 30 minutes at 4°C, and subsequently centrifuged at 4000× g for 10 minutes. The supernatant was removed and the resulting pellet was reconstituted by sonication for 15 min in 10 mL water/acetonitrile (1∶5, v/v). The resulting suspensions were chilled for 30 min at 4°C, and then centrifuged at 8000× g for 10 minutes. The combined supernatants were evaporated to dryness under a stream of nitrogen at room temperature. The remaining residues were reconstituted in water (0.2 mL) then transferred into a 1.5 mL ultracentrifuge tubes (Beckman, Krefeld, Germany). The supernatants obtained were separated from the pellets then transferred into a HPLC vial, from which the tare weight was previously determined. The solutions were concentrated under nitrogen to approx. 100 µL. After addition of 25 µL of acetonitrile, the concentrates were completed with water up to approx. 0.25 g and aliquots of 80 µL were injected on the HPLC component of the automation platform described below.

Urine for each subject was pooled across the collection period of 0–96 hours. Aliquots of 1 mL were centrifuged at 10000× g for 15 minutes and stored at −20°C until HPLC analysis. Aliquots of 100 µL of supernatants were directly injected on the HPLC component of the automation platform described below.

Feces homogenates (diluted with water) of each subject were pooled across the collection period of 0–96 hours. An aliquot of approx. 0.6 mL of each pool were mixed with 10 mL acetonitrile. The suspensions were stirred for 60 minutes at room temperature followed by centrifugation for 15 minutes at 10000× g. After removal of the supernatants, the residues were extracted a second time with 1 mL water and 10 mL acetonitrile, using the same procedure. Both supernatants were combined, and evaporated to dryness under a stream of nitrogen at room temperature. The residues were reconstituted by addition of acetonitrile (100 µL) and water (900 µL). Aliquots of 100 µL were injected on HPLC component of the automation platform described below.

### Instrument Methods

An Agilent 1200 HPLC (Agilent Technologies, Waldbronn, Germany) equipped with two binary pumps, Degasser, ALS Thermostat, a CTC PAL Autosampler (CTC Analytics, Zwingen, Switzerland) was used for HPLC method coupled to PlateCrane (Hudson Robotics Inc, Springfield, USA).

#### Hplc parameters

Guard column: Atlantis dC18, 20×2.1 mm, 3 µm particles, (Waters, Baden-Dättwil, Switzerland).

Analytical column 1: Atlantis dC18, 150×2.1 mm, 3 µm particles, (Waters, Baden-Dättwil, Switzerland).

Temperature of guard column and analytical column: 40°C.

Sample injection (default): 20 to 100 µL injected via a 200 µL sample loop.

Flow: 350 µl/min.

Mobile phase A: ammonium formate 20 mM, with 0.1 % of formic acid; pH 3.6 B: acetonitrile.

HPLC-Gradient: 0–35 min 10–25% B, 35–60 min 25–50% B, 60–65 min 50–90% B, 65–70 min 90% B, 70–72 min 90–10% B, 72–90 min 10% B.

An Acquity UPLC system (Waters, Milford, USA), equipped with two binary pumps, column manager, Degasser, PDA detector, and a CTC PAL Autosampler (CTC Analytics, Zwingen, Switzerland) was used for UPLC method.

#### Uplc parameters

Guard column: Acquity UPLC HSS T3 C18 Van Guard Pre-Column, 2.1×5 mm, 1.8 µm particles, (Waters, Baden-Dättwil, Switzerland).

Analytical column: Acquity UPLC HSS T3 C18, 150×2.1 mm, 1.8 µm particles, (Waters, Baden-Dättwil, Switzerland).

Temperature of guard column and analytical column: 40°C.

Sample injection (default): 20 to 100 µL injected via a 200 µL sample loop.

Flow: 500 µl/min.

Mobile phase A: ammonium formate 20 mM, with 0.1 % of formic acid; pH 3.6 B: acetonitrile.

Gradient: 0–17 min 10–25% B, 17–30 min 25–50% B, 30–32 min 50–90% B, 32–35 min 90% B, 35–36 min 90–10% B, 36–45 min 10% B.

#### Mass spectrometry parameters

Both LC Systems were in line with a Synapt Quadrupole-time-of-flight tandem mass spectrometer (Waters, Manchester, UK) and a GX271 fraction collector (Gilson, Villiers-le-Bel, France) with Plate Crane EX (Hudson Robotics Inc., Springfield, USA). The Agilent LC system was controlled by ChemStation B.03.02 (Agilent Technologies, Waldbronn, Germany) The UPLC and MS Systems were controlled by MassLynx V4.1 (Waters, Manchester UK). The fraction collector was controlled by Trilution V1.4 LCOK8197 (Gilson, Villiers-le-Bel, France), and the Plate Crane by SoftLinx V3.10 (Hudson Robotics Inc., Springfield, USA).

After the chromatography the effluent was split into a ratio of 1∶8 with the smaller portion directed into the electrospray LC-MS interface and the bigger part was used for offline radio detection (metabolites pattern).

MS spectra with accurate mass measurement for [^14^C]-NVP123 were obtained by LC-MS on Synapt Q-TOF (Waters, Manchester UK) in positive electrospray mode. Capillary voltage 3 kv, Cone voltage 20 V, scan range m/z 100–1000. MS spectra were acquired in MS^E^ mode. The reference channel of the LockSpray interface was operated with a solution of reserpine (575 ng/mL) in acetonitrile at a flow rate of 5 µL/min. During data acquisition from the reference channel, the cone voltage was set to 40 V. The [M+H]^+^ ion of reserpine at *m/z* 609.2812 was used as lock mass for recalibrating the spectra to obtain exact mass data.

The resulting tab-delimited data were converted into chromatograms and integrated using an internal Novartis Nitisc program (Version V12). Standard chromatographic peak integration software would also be suitable for these purposes.

#### Set-up of PlateCrane robot and Gilson fraction collector

The PlateCrane robot and Gilson fraction collector with custom programmed firmware provided by Gilson were mounted on a movable table with lockable castors. The robotic arm of the PlateCrane was programmed and synchronized so that it does not to collide with arm of Gilson fraction collector. Also, the positioning of Luma microplates in Gilson fraction collector was programmed to ensure the most efficient sampling. The entire analytical system consisted of a LC-MS with CTC autosampler, PlateCrane and Gilson fraction collector. The CTC autosampler triggered the start of the LC-MS and the fraction collector. The PlateCrane was initiated and controlled by the Gilson Trilution software. Custom programming scripts were created by Hudson and Gilson engineers. The PlateCrane should be placed in close proximity to the LC-MS system to minimize chromatography delay and peak broadening. An illustration and photo of instrumental set-up are shown in Figure 1 and 2.

For the PlateCrane, a maximum of 10 “stack columns” are available (Figure 1). Each stack column can hold up to 24 Luma plates. Stack columns 1–5 are reserved for “empty” plates and stack columns 6–10 for “filled” plates. Based on current set up, a maximum of 120 plates can be automatically collected with PlateCrane. The chronological sequence for picking up and placing plates from stack columns can be customized and programmed according to experiment. In principle, the system could almost double its capacity by reprogramming to accommodate 216 plates. The 9 stack columns would be loaded with empty plates while one stack column would contain no plates. The one empty stack column would be loaded with filled plates, which would eventually generate another stack column with no plates. This has not been done as no more 120 plates have been exceeded in an automation sequences to date.

The PlateCrane and fraction collector software was programmed that for both 96 or 384 well Luma plates where a maximum of 8 plates can be used within a single chromatographic run. As a note, the fraction collector can hold a maximum of 2 Luma plates on deck in position 1 and 2 (Figure 1)

#### A chronological outline describing the automation process: (Figures 3 and 4)

Before any sample injection, all methods and sequences need to be programmed on the HPLC, Gilson fraction collector and MS instruments.

1. The Gilson fraction collector is first initiated from software (Trilution) and commands the PlateCrane robotic arm to pick up a 96 or 384 well plate from stack 1 and place it in position of plate 1. After completion of this task, the Trilution software is ready to receive start signal via contact closure from autosampler. The MS sequence list is then initiated from the MS software (MassLynx). The MS system is now awaiting a trigger signal from the HPLC autosampler.
2. Once sample has been injected by the HPLC autosampler, a contact closure signal simultaneously triggers MS acquisition, UV detector (if programmed), and the fraction collector, which has now started HPLC eluant collection. Ten seconds after the initial fractions were collected, the fraction collector requests a “Ready” signal from PlateCrane. The Platecrane responds “Ready Yes”. Then robotic arm picks-up a second empty Luma plate from stack 1 and places it on deck of fraction collector in position 2.
3. After collection of first Luma plate has been completed, fraction are automatically continued to be collected on second plate. Ten seconds after starting collection on second Luma plate, the PlateCrane requests pick-up of filled plate number 1 from deck of fraction collector. The robotic arm picks up filled plate number 1 and places it in stack 6. The PlateCrane automatically picks up an empty plate from stack 1 and places it on the deck of fraction collector in position 1. After the second Luma plate in position 2 has been filled completely, fraction collection continues automatically on third plate in position 1 on the deck of fraction collector. For changing the third and the fourth plate within this run, the same process is followed as described in step 3.
4. After last Luma plate has been collected the robotic arm places the filled plate in stack 6 and new plate from stack one is placed in position 1 on deck and robotic arm goes to “Home” position and awaits the trigger signal from next injection.
5. For each injected sample in the programmed sequence, steps 2 and 3 are repeated. Throughout the automation, the PlateCrane takes empty Luma plates in sequential order from stack columns 1–5, and places subsequently filled plates in the empty stack columns 6–10 respectively.
6. After last plate in the sequence has been collected signal “End sequence” is sent to PlateCrane. The PlateCrane then places last plate in stack and Trilution software does not demand for new plate. The robotic arm moves in “Home” position.
7. After collection of plates and the “automation” process is complete. The plates are either under vacuum centrifugation using a Speedvac AES1010 (Savant Instruments Inc., Holbrook, USA), or air dried in fume hood for 2 h. The dried plates are heat sealed and loaded on Topcount instrument for radioactivity measurements.

#### Topcount radioactivity measurements

The dried scintillation plates were analyzed in a microplate scintillation counter (TopCount NXT; Packard Instruments, Meriden, CT, US). In order to optimize TopCount performance, counting heads (photomultipliers) were reconditioned and then closely matched with one another in terms of tolerances and specifications (TopLab GmbH, Switzerland). The matched heads were then (re)installed in the TopCount instrument. Counting times were between 3×5 min to 3×40 min, depending on the amounts of radioactivity injected. Counts were monitored during the three counting periods were averaged unless one of the three measurements was an outlier in the positive direction (possibly due to an electrostatic discharge), in which case only the counts from the two remaining counting periods were averaged. Moreover, a correction was made for the different background levels of the 12 photomultipliers of the microplate scintillation counter.

## Results and Discussion

Automation technologies and processes are well established and specifically tailored for high throughput ADME-related screening during the drug discovery phase. However, high volume automation platforms often do not translate well for applications which are not screening type assays, nor are high volume platforms designed for these purposes. Thus, a clear gap exists for such studies which do not require specialized high volume automation technologies, yet these studies would still clearly benefit from automation. *In vitro*, preclinical *in vivo* and human ADME studies conducted during the drug development phase which have a metabolite identification component coupled with radioactivity measurements are a good example.

To address this gap and increase throughput for metabolite identification in ADME studies using Topcount measurement, the PlateCrane was coupled to fraction collector as described in the experimental section. This “hybrid” system allows continuous changing of fraction collected plates (maximum 120 plates) which drastically reduced manual 96-and 384 well plate collection and handling. A schematic description of system configuration is shown in Figure 1 and a photo of the system is shown in Figure 2. The automated plate changing process and sequence is shown in Figure 3 and 4. These steps are detailed within the experimental section.

The PlateCrane and Gilson fraction collector components are mounted on movable table with lockable castors, which allows flexibility of use in different labs. This “hybrid” system allows continuous changing of fraction collected plates. Chromatographic sample output can be increased up to 10 (and more depending run time) samples per day on a 24/7 basis. This technology was successfully implemented and supported a human ADME study for a designated Novartis development compound ([^14^C]-NVP123) that required metabolite profiling and identification.

A general workflow process for metabolite identification with and without PlateCrane is illustrated in Figure 5. The manual plate changing step denoted in Figure 5 is one rate limiting step in the process. Furthermore, Table 1 highlights time differences for different steps in process with and without PlateCrane. For example, an analysis of 8 samples with Platecrane results in a time savings of 4 days as compared to without PlateCrane. Moreover, the number of samples injected within one day could be increased up to 10 fold as this system is able to constantly change plates throughout the night and over the weekends while left unattended (Table 1). Overall, the net benefit was about a 32% increase in productivity when all steps were counted in the workflow (Figure 3 & Table 1).

A typical reconstructed HPLC metabolic profile of [^14^C]-NVP123 in human plasma acquired with described automation set-up is shown in Figure 6. The gross sample analysis time for this complex ADME study was completed 4 months sooner compared to similar studies using conventional methods (without PlateCrane). Studies using conventional methods currently take about one year for completion. Thus, the time savings from this set-up has a substantial positive impact on the development program of drug. Integration of UPLC-MS with PlateCrane and Gilson Fraction collector exemplifies the flexible nature of this evolving platform. Chromatographic run times could be shortened from 90 minutes down to 30 min. The narrow peak widths and longer counting times available from UPLC combined with TopCount provides a more sensitive method of profiling drug metabolites in complex biological samples, particularly when samples contain low concentrations of drug-related material. Figure 7 shows a chromatogram of [^14^C]-NVP123 in dog urine. The narrow peak widths and shorter chromatographic run times available from UPLC combined with TopCount provide a more sensitive method of profiling drug metabolites in complex biological samples, particularly when samples contain low concentrations of drug-related material.

In addition, MS spectra for metabolite identification data can be collected in parallel, i.e. the same way as done for HPLC-MS using a RFD. Note, the MS data generated for structure elucidation using PlateCrane automation was compared and obviously confirmed to be identical with the MS data generated using the prior method of manual injection (MS data or structures not shown). Figure 8 shows the structure elucidated by mass spectrometry for [^14^C]-NVP123, M1/M2, M3 and M7 metabolites. All the metabolite structures listed in the chromatograms (Figure 6 & 7) have also been elucidated by mass spectrometry and then confirmed using co-chromatography and MS with authentic standards where applicable. Figure 9 shows a biotransformation overview resulting from the example study, which used the robotic plate handling platform, HPLC / UPLC system, mass spectrometer and the automated fraction collector.

The focus of Figures 6, 7, 8, and 9 is to fully highlight the representative types of data output generated from this combination platform (automated fractionation and plate handling, chromatography, and MS structural characterization with proposed fragmentation schemes). Full structures will be shown in a separate publication which will be dedicated to interpretation and results of the project itself, and not the automation platform. Thus, partial structures fulfilled these purposes and were shown for this reason.

### Conclusions

This novel linked automated system significantly increases throughput for preclinical and clinical ADME studies. The plate changing process has now been automated, and time for manual sample manipulation has been significantly reduced. This technology drastically reduced manual 96-well/384-well plate collection and handling as a maximum of 120 plates could be collected automatically. Sample analysis of [^14^C]-NVP123 for the presented human ADME study including data analysis and reporting was completed faster, i.e. in about 8 months as compared to one year. Throughput in preclinical and clinical ADME studies has clearly benefited from this new technology, and this has positive impact on accelerating drug development studies. In summary, a major limiting step or “bottle neck” has essentially been eliminated from the process. A remaining limiting step is long sample counting times that may be required by MSCs. A potential future application of this automation platform could also be the chromatographic separation and isolation of metabolites on a preparative scale.
